# Immunological characterization of recombinant *Salmonella enterica* serovar Typhi FliC protein expressed in *Escherichia coli*

**DOI:** 10.1186/2191-0855-2-55

**Published:** 2012-10-15

**Authors:** Gaurav Jindal, Rupinder Tewari, Ankur Gautam, Satish K Pandey, Praveen Rishi

**Affiliations:** 1 Department of Biotechnology, Panjab University, Chandigarh, 160014, India; 2 Department of Microbiology, Basic Medical Sciences Block, Panjab University, Chandigarh, 160014, India

**Keywords:** Outer membrane proteins (OMPs), *Salmonella enterica* serovar Typhi, Flagellin (FliC), Immunogenicity, Inflammation, Thermal hyperalgesia

## Abstract

Like any other enteric pathogen, *Salmonella* also encounters acidic stress in the stomach as well as within the host macrophage milieu. However, the pathogen is reported to combat this stress through acid tolerance response (ATR), expressing a number of genes and eventually the proteins. Recently, an acid induced outer membrane phenotype encoded by *fliC* gene in *Salmonella enterica* serovar Typhi has been identified. In the present study, *fliC* gene was cloned to study its biological implications. The recombinant FliC (rFliC) protein was observed to stimulate the production of antibodies. These antibodies could also recognize the FliC protein (antigen) in the clinical samples i.e. blood samples from typhoid patents as well as healthy blood samples spiked with serovar Typhi. Moreover, the rFliC also reacted with the sera from patients suffering with typhoid fever indicating its *in-vivo* immunogenicity. *Ex-vivo* study revealed that rFliC has the potential to stimulate the macrophages to generate higher levels of inflammatory mediators such as malondialdehyde (MDA) and nitrite. The inflammatory potential of FliC was also confirmed *in-vivo*, by the paw oedema test as well as by flicking response of the inflamed paw indicating hyperalgesia occurring during inflammatory response. The findings of the present study indicate that acid induced FliC might be one of the factors enhancing the virulence of serovar Typhi under the host acidic conditions and may prove to be helpful in designing the prophylactic measures.

## Introduction

*Salmonella* pathogenicity is multifactorial and the expression of various virulence determinants has been reported to differ under *in-vivo* and *in-vitro* situations (Chanana et al. 2007a, b; Cruz et al. 2010 and Diacovich et al. 2010). Therefore, there is a renewed interest in understanding the behavior of the pathogens in different environments of the host like low pH, elevated temperature, changes in osmotic strength, presence of cationic peptides as well as the availability of ions and nutrients.

*Salmonella* has been reported to combat inorganic acidity in the stomach as well as in the intracellular milieu of macrophages through acid tolerance response (ATR). A number of genes including Pho*P/Q* regulon, *fur* gene (iron stress), Rpo*S* and *adi* operon regulating arginine decarboxylase in *Salmonella* (Bearson et al. 1998 and Kieboom et al. 2006) have been identified for combating acid stress. However, the paucity of information on stress induced proteins of serovar Typhi, the causative agent of typhoid fever is surprising in view of *Salmonella* pathogenicity being multi-phasic and multi-factorial. Very little is known about the phenotypic expression of the pathogen in the ever changing environment of the host with respect to outer-membrane proteins which first come in contact with the host.

The outer-membrane proteins (OMPs) of bacteria function as a dynamic interface between the bacterium and its surrounding environment and are involved in regulating the transport of nutrients and bactericidal agents. However, functions of OMPs induced under the host conditions have not been specified particularly in serovar Typhi. Recently, *fliC* gene encoding an acid induced outer-membrane protein (AIP) has been identified (Jindal et al. 2011). Prompted by this observation, *fliC* gene of serovar Typhi was cloned to assess the *in-vivo* immunogenic and inflammatory potential of FliC phenotype in the present study.

## Materials and methods

### Reagents

Luria-Bertani (LB) medium (Hi-media, India) was used for bacterial culture. Chemicals and antibiotics were purchased from Sigma-Aldrich, USA and IPTG (Isopropyl-β-D-thiogalactopyranoside) was purchased from USB Corp., USA. All restriction and modifying enzymes for manipulation of DNA were obtained from MBI Fermentas, Germany. Chromatographic columns and molecular weight markers for gel filtration chromatography were purchased from GE Healthcare Biosciences, Piscataway, USA with the exception of nickel nitrilotriacetic acid (Ni^2+^-NTA) -agarose which was purchased from Qiagen, USA. Custom oligonucleotides were purchased from Sigma Genosys (Bangalore, India).

### Bacterial strains, plasmids and culture conditions

Standard strain of *Salmonella enterica* serovar Typhi Ty2 (strain DBL-8, David Bruce Laboratory, East Everleigh, near Marlborough Wiltshire) was initially procured from Central Reasearch Institute, Kasauli, India. pET28c (+) plasmid, *E. coli* DH5α and *E. coli* BL21 (DE3) (Novagen, USA) were used as expression vector, for propagation of plasmid and as an expression host, respectively. *E. coli* strains were grown aerobically at 37°C in LB medium and the recombinant *E. coli* strains were cultivated in the same medium (Sambrook et al. 2001) containing 50 μg ml^−1^ kanamycin. The medium was inoculated with 1% of 10–12 h seed culture and incubated under shaking conditions at 37°C and 150 rpm.

### Clinical samples

Sixteen clinical samples (13 Widal positive sera and 3 blood culture positive blood samples) were collected from various hospitals in the city. 4 blood samples collected from apparently healthy individuals were spiked with different concentrations of serovar Typhi cells.

### Animals

BALB/c mice (18–22 g) (either sex) were procured from Central Animal House, Panjab University, Chandigarh (India). The animals were housed under standard conditions of light and dark cycle with free access to feed (Ashirwad Industries Pvt Ltd, Ropar, India) and water *ad-libitum*. Care and use of animals was in accordance with the guidelines of the Institutional Animals Ethics Committee, Panjab University, Chandigarh (India).

### Extraction and characterization of acid stress induced OMPs

Acid stress induced OMPs were extracted from bacterial cells grown under inorganic acid stress conditions (pH 4.5, adjusted with 1 N HCl) according to the method described earlier (Chander et al. 2004) and the protein profile was analyzed by SDS-PAGE.

### PCR amplification and construction of recombinant plasmid

The *Salmonella enterica* serovar Typhi *fliC* gene was PCR amplified using Hi-Fidelity^TM^ DNA polymerase (MBI Fermentas, Germany) and a set of modified primers designed by using DNASTAR (Lasergene Inc., USA).

Primer F- (5^′^-TATATCCATGGCACAAGTCATTAATACAAACAGCCTGT-3^′^ containing a *Nco*I-engineered restriction site).

Primer-R (5^′^-ATTACTCGAGACGCAGTAAAGAGAGGACGTTTTGC-3^′^ containing a *Xho*I-engineered restriction site).

The resulting *fliC* PCR product was double digested by *Nco*I and *Xho*I (MBI Fermentas, Germany), then cloned in pET-28c (+) plasmid digested by the same enzymes to form the expression plasmid pETFliC with a kanamycin resistant selectable marker. *E. coli* DH5α transformants grown overnight on LB plates containing kanamycin (50 μg ml^−1^) were screened (Sambrook et al. 2001). The homogeneity of cloned *fliC* was confirmed by DNA sequencing which was performed using sequencing primers for pET28c(+) vector by the fluorescent dideoxyterminator method using an ABI 3100 capillary sequencer (ACTG Inc., USA).

### Expression and purification of rFliC in *E. coli*

A single colony of *E. coli* BL21 (DE3) harboring recombinant pET28c (+) *fliC* plasmid was inoculated in 5 mL LB broth (Hi-media, India) containing kanamycin (50 μg/mL) and was grown overnight at 37°C. 1% (v/v) of overnight grown culture was inoculated in 400 mL LB medium containing kanamycin (50 μg/mL) and cells were allowed to grow to an optical density (OD_600_) of 0.5-0.6 at 37°C. The expression of target protein was induced by adding isopropyl-1-thio-β-d-galactopyranoside (IPTG) (final concentration varies from 0.1 to 1.0 mM) under different conditions (15°C-37°C, 4–8 h, 200 rpm).

The 8 h induction cultures were harvested by centrifugation for the purification of recombinant protein. Wet cells were re-suspended in 20% (v/v) lysis buffer (50 mM Tris–HCl pH- 8.0, containing 300 mM NaCl, 2 mM β-mercaptoethanol, 1 mM phenylmethylsuphonyl fluoride and 10% glycerol) and cells were disrupted by sonication. The debris was pelleted by centrifugation at 13,400 × *g* for 15 min at 4°C and re-suspended in the lysis buffer (pH-8.0) containing 8 M Urea. The debris was removed by centrifugation at 15,000 × *g* for 20 min at 4°C. The supernatant was added to a Ni^2+^-NTA resin column (1 ml bed volume) equilibrated with lysis buffer and was allowed to bind slowly. After being washed with 4–5 ml of the lysis buffer containing 20 and 40 mM imidazole, the target protein was eluted using elution buffer (the lysis buffer with 300 mM l^−1^ imidazole) and 1.5 ml fraction was collected. All the fractions were pooled and dialyzed thoroughly against 50 mM Tris–HCl containing 150 mM NaCl, 1 mM DTT (dithiotheritol) and 10% glycerol. The purified rFliC was then used for further experiments. Protein concentration was determined by Bradford method (Bradford et al. 1976), using Bovine Serum Albumin (BSA) as standard.

Though *fli*C gene was cloned and expressed in *E. coli* successfully, but further analysis revealed that most of the rFliC was present in inclusion bodies or insoluble fraction. Changing poly-histidine tag from N-terminal to C-terminal, lowering of incubation temperature and IPTG concentration for induction followed by dialysis resulted in the production of rFliC in soluble fraction which is in concordance with the findings of Shafiani et al. (2005).

### Biological implications of rFliC

#### In-vivo expression of rFliC

The *in-vivo* immunogenicity of rFliC was assessed qualitatively as well as quantitatively. For this, anti-serum to rFliC was raised in New-zealand rabbits and recognition of antigen by the raised antiserum was evaluated by ELISA as described earlier (Chander et al. 2004). Briefly ELISA plates were precoated with 100 μl Poly-L-lysine (0.1 M) (Sigma) in phosphate buffer saline (PBS), pH 7.4 and incubated for 1 hour at room temperature. The plates were washed three times with PBS. 100 μl of different concentrations of Fli C protein (0 to 100 μg/ml) or healthy blood sample spiked with cultured serovar Typhi as well as typhoid patients’ blood samples were added to respective wells of ELISA plate. The coated plates were incubated overnight at 4°C, and then washed three times with PBS containing 0.05% Tween 20 (PBST). The antiserum against FliC antigen was diluted (1:10000) in PBS-M (phosphate buffer containing 0.1% skimmed milk) and 100 μl was added in each well. The plates were then incubated for 1 hour at 37°C. After washing three times with PBS-T, secondary antibody (alkaline phosphatase conjugated goat anti-rabbit IgG) (Sigma) was diluted (1:10000) in PBS-M, and 100 μl was added to each well and again the plates were incubated for 1 hour at 37°C . The plates were washed again, and subsequently 100 μl of p-nitrophenylphosphate was added to each well. Thereafter, the plates were incubated at 37°C for 20 min and absorbance was measured at 405 nm. This, quantitative evaluation for reactivity of anti-FliC antibody was assessed by ELISA using (i) rFliC antigen as such as well as (ii) patient blood samples or healthy blood samples spiked with different concentrations of clinical isolates of serovar Typhi. Qualitative evaluation for assessing the reactivity of rFliC with the sera from patients suffering with typhoid fever (Widal positive) was performed by Western blotting as described by us earlier (Jindal et al. 2011).

### Extraction of peritoneal macrophages and their interaction with Salmonella and/or rFliC

Murine peritoneal macrophages were isolated (Preet et al. 2010) and cell viability was checked using 0.2% trypan blue dye. Following extraction, peritoneal macrophages were interacted with *Salmonella enterica* serovar Typhi (at multiplicity of infection (MOI) 1:100) and with protein (7.5 μg/10^6^ of macrophages) extracted under normal and stress conditions in tissue culture plate at 37°C for 6 h and 16 h respectively. The dose of protein and time of interaction was optimized after thorough standardization using different concentrations of normal OMPs, acid induced OMPs and rFliC at various time intervals.

### Assessment of *ex-vivo* inflammatory potential of rFliC

#### Determination of malondialdehyde (MDA) Level

The amount of MDA formed (a measure of lipid peroxidation) in the culture supernatant of macrophages was assayed by the reaction with thiobarbituric acid (TBA) (Wills et al. 1966). The results were expressed as nanomoles of MDA per mg of protein, using the molar extinction coefficient of chromophore (1.56 x 10^5^ M^-1^ cm^-1^).

### Determination of nitrite levels

The amount of nitric oxide in cell free supernatant was determined by Griess reaction (Green et al. 1982). The assay is based on the propensity of nitric oxide, oxidized to nitrate and nitrite under physiological conditions as described by Bharrhan et al. (2011). The nitrite levels in all the samples were quantified according to the standard graph of sodium nitrite.

### Assessment of *in-vivo* inflammatory potential of rFliC

#### Paw oedema test

To assess the *in-vivo* inflammatory potential of rFliC protein by the oedema test qualitatively as well as quantitatively, mice were divided into the following groups, each comprising of six mice. Each mouse was injected intradermally with 0.1 mL of the following preparations in the dorsal foot pad of the left paw (Rishi et al. 2008), (i) 1% (w/v) of carrageenan (Hainan Kaiyang Trade Co. Ltd, China) (positive control); (ii) 7.5 μg of OMPs of *Salmonella enterica* serovar Typhi grown at pH-7.0; (iii) 7.5 μg of acid-induced OMPs of serovar Typhi grown at pH-4.5; (iv) 7.5 μg of rFliC. Normal saline injected in the right paw of each mouse served as a negative control. All the mice were observed at regular intervals up to 3 h for inflammation. Quantitative assessment of oedema was done using an plethysmometer, which is instrument containing mercury. In brief, the paws of test animals were marked above the tibiotarsal junctions to ensure that every time paw was dipped to the same level in the mercury column of plethysmometer, which was earlier described by our group Choudhary et al. (2005).

### Hyperalgesia test

After 3 h, flicking response of the inflamed paw was assessed using the paw immersion (warm water) test. The animals were marked on both the hind paws (right and left), just beyond the tibiotarsal junction so that the mouse paw was dipped to the same level in the water bath every time. The paw was immersed in the warm water bath (47 ± 0.5°C) until signs of struggle (paw withdrawal) were observed. The paw flicking response in terms of time in each of the above groups was recorded.

### Statistical analysis

Results were expressed as mean ± standard deviation (SD). The inter group variation was assessed using one way analysis of variance (ANOVA) followed by Fischer’s LSD test. Statistical significance of the results was calculated at least at p< 0.05.

## Results

### Characterization of acid stress induced OMP

The over-expressed acid induced protein (AIP) was characterized using in-gel trypsin digestion followed by peptide mass fingerprinting (MALDI-TOF) and was identified as a major structural component of flagella encoded by serovar Typhi *fliC* gene as described by us earlier (Jindal et al. 2011).

### Cloning of serovar Typhi fliC gene

**Figure 1:**
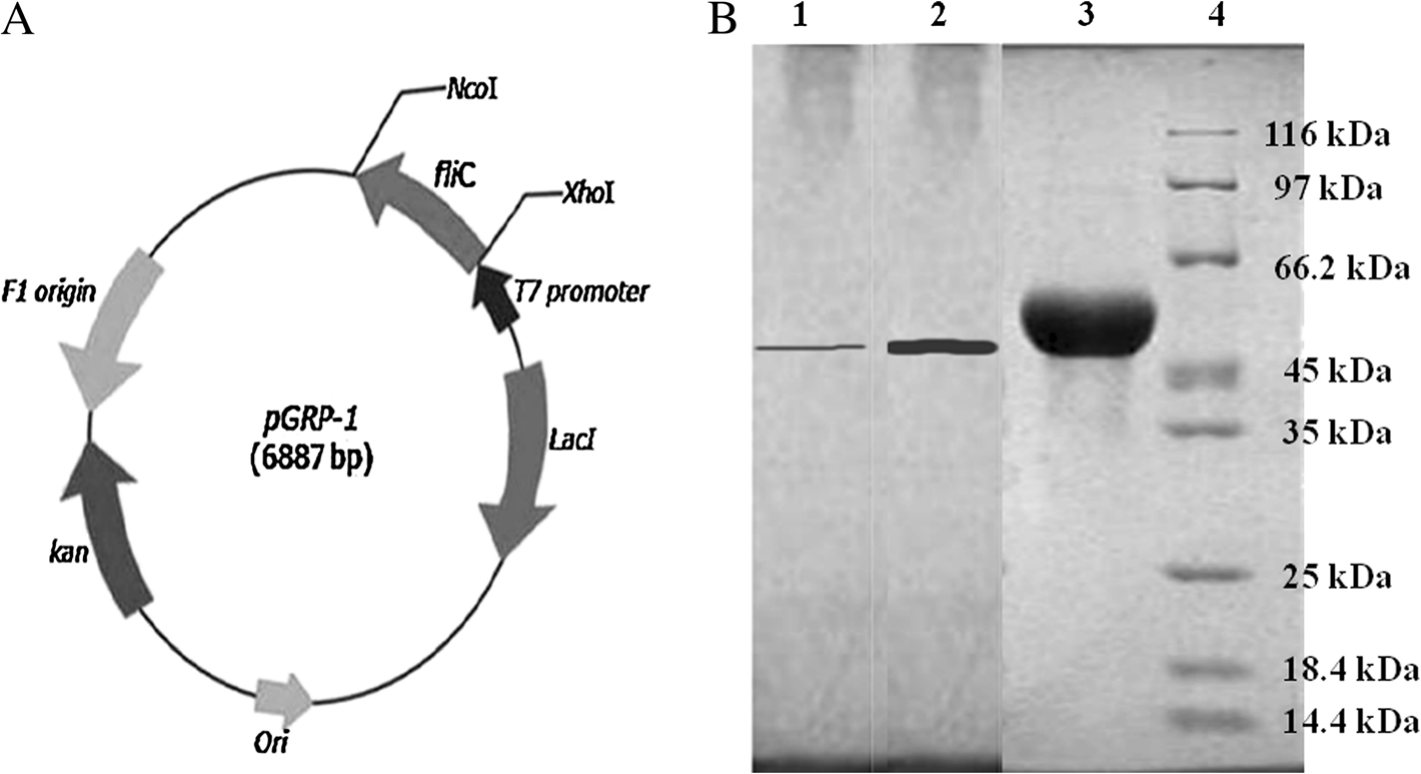
**Schematic presentation of the pET28c+ vector construct (6887 bp) containing the *fli *C gene responsible for the expression of rFliC designated as pGRP-1. **Figure 1B: Expression of the purified rFliC by SDS-PAGE. Lanes-1, 2 &3: A single band of purified rFliC (different amount of protein) observed after Ni^2+^-NTA affinity chromatography of cell lysate of IPTG induced *E. coli *BL21 (DE3) cells; & 4: Standard Protein Molecular Weight Marker (broad range).

The ORF of *Salmonella enterica* serovar Typhi cloned *fliC* was found to consist of 1521 bp which encoded a polypeptide containing 506 amino acids with a predicted molecular mass of 53.2 kDa and a theoretical iso-electric point of 4.1. The homogeneity of cloned *fliC* was confirmed through DNA sequencing by primer walking. Compared with most other sequenced *fliC* in the BLAST databank, the deduced amino acid sequence of FliC was found to be similar to FliC from serovar Typhi Ty2 (100% identity) (Parkhill et al. 2001). The pET*fliC* plasmid construct was designated pGRP-1 (Figure 1A).

### Expression and purification of rFliC

The expression of the recombinant FliC was analyzed by SDS-PAGE. A clear band around 53 kDa was observed from *E. coli* BL21 (DE3) harboring pGRP-1 after 8 h induction at 30°C using 0.4 mM IPTG concentration (Figure 1B). The molecular mass of the rFliC was 53.2 kDa consisting of 1521 amino acid residues of FliC and 18 amino acid residues of C-terminal fusion segment. It contained the His_(6)_-affinity tag and a unique thrombin cleavage site.

The distribution of the expressed target protein in total cell lysate, soluble fraction and in the insoluble fraction was also analyzed by SDS-PAGE (data not shown). The major proportion of target recombinant protein in cell lysate was found to be accumulated in the precipitates and only a trace amount was observed in the soluble fraction. The recombinant His_6_-FliC from the precipitates was shown to have recovered in the soluble form after dialysis.

### Purification of rFliC

The thick band was separated on SDS-PAGE (Figure 1B, lane-3) by means of the affinity chromatography with Ni^2+^-NTA resin. The recombinant FliC was found to be homogenous as indicated by a single protein band on SDS-PAGE. The band of approximate 53 kDa corresponded to the rFliC. The yield of purified rFliC protein was in the range of 6–10 mg/L.

### Biological implications of rFliC

#### In-vivo immunogenicity of rFliC

**Figure 2:**
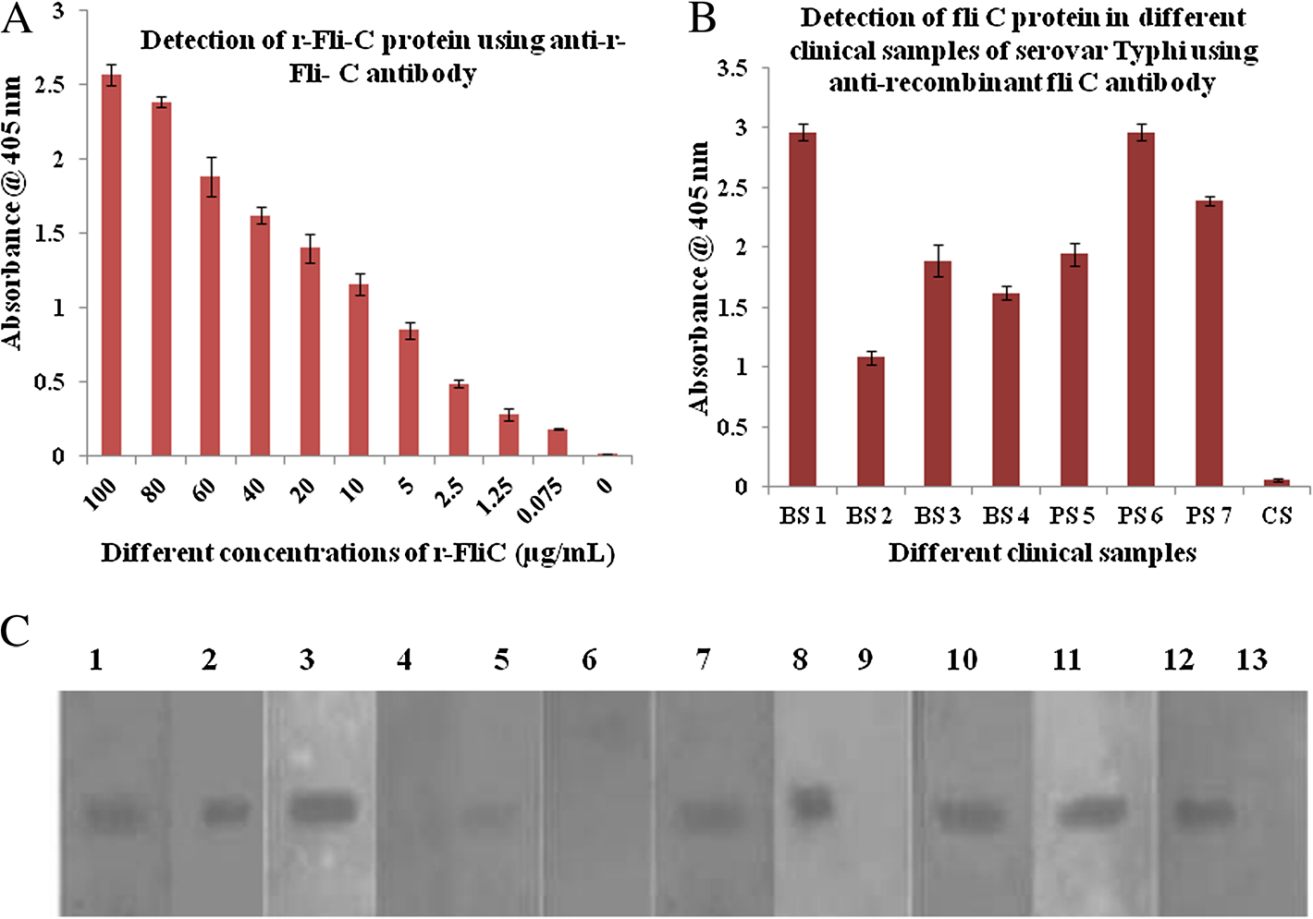
**Detection of recombinant FliC protein of serovar Typhi (r-FliC) by ELISA using anti-rFliC antibody. **This shows the detection of FliC in different concentrations indicating the FliC variability. Figure 2B: Detection of FliC antigen in clinical samples. Representative bars shows healthy blood samples spiked with cultured serovar Typhi (BS1 to BS4) and patient’s blood samples (PS5 to PS7) using anti-r-FliC antibody by ELISA. NC is negative control i.e. unspiked healthy blood sample. Figure 2C: Western blots analysis showing the reactivity of rFlic with patients’ sera. Lanes-1-3,5,7,8 &10-12: Ni^2+^-NTA purified rFliC of serovar Typhi showing reactivity with typhoid patient (Widal positive) serum on PVDF membrane; Lane-4: Ni^2+^-NTA purified rFliC of serovar Typhi showing no reactivity with patients serum.

The titre of anti-rFliC antibodies in the hyper-immune serum was found out to be 1: 10000 indicating the immunogenic potential of rFliC. An antibody titer is defined as highest dilution of the antibody that gives positive result with the minimum amount of antigen. The reactivity of anti-rFliC antibodies with different concentrations of FliC of serovar Typhi has been shown in Figure 2A. This clearly shows that the raised antibodies could detect rFliC antigen to the level of 1.25 μg/mL. Further, raised antibodies could also detect FliC in the patient’s blood samples or healthy blood samples spiked with cultured serovar Typhi (Figure 2B). This figure also shows the absence of the antigen in unspiked healthy blood sample. Reactivity of rFliC with the sera of typhoid patients (Widal-positive) revealed its *in-vivo* immunogenicity. Out of 13 typhoid patients sera tested, 9 sera were recognized by rFliC protein (Figure 2C). In contrast, none of the six normal sera from apparently healthy individuals reacted with this protein.

### Effect of rFliC on macrophage inflammatory molecules (*Ex-vivo*)

#### MDA levels

**Figure 3:**
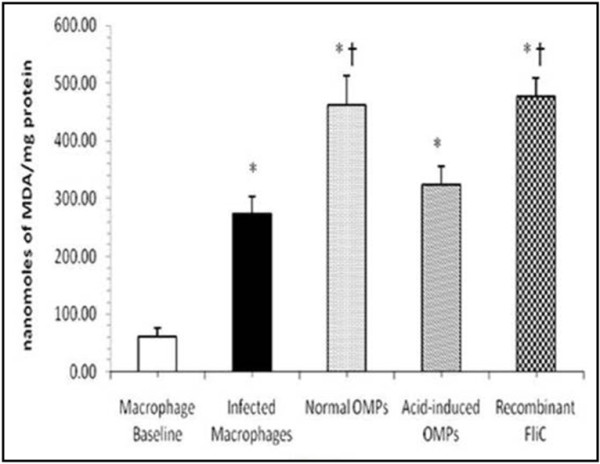
**MDA levels in the culture supernatants of macrophages incubated with rFliC (10 μg/mL), acid-induced OMPs (10 μg/mL), normal OMPs (10 μg/mL) and serovar Typhi cells. **Values are expressed as mean ± S.D. ^*^p<0.001 vs. not incubated macrophages (macrophage baseline); ^†^p<0.01 vs. macrophages infected with serovar Typhi cells.

On interacting the macrophages with the rFliC (Figure 3) it was found that the protein could stimulate the macrophages to produce significantly higher MDA levels (p<0.001) as compared to that of the macrophages interacted with *Salmonella enterica* serovar Typhi cells (p<0.05), OMPs of serovar Typhi grown under normal conditions and with OMPs of serovar Typhi grown under acid stress conditions (p<0.05).

### Nitrite Levels

**Figure 4:**
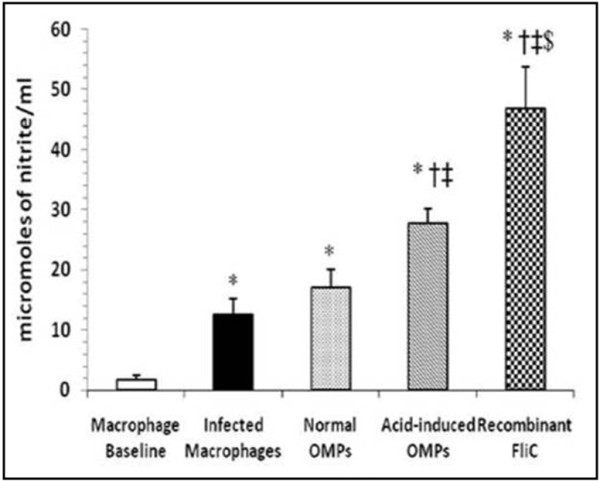
**Nitrite levels in the culture supernatants of macrophages incubated with rFliC (10 μg/mL), acid-induced OMPs (10 μg/mL), normal OMPs (10 μg/mL) and serovar Typhi cells. **Values are expressed as mean ±S.D. ^*^p<0.001 vs. not incubated macrophages (macrophage baseline); ^†^p<0.001 vs. macrophages infected with serovar Typhi cells; ^‡^p<0.01 vs. macrophages interacted with normal OMPs; ^$^p<0.001 vs. macrophages interacted with acid-induced OMPs.

The rFliC significantly (p<0.001) increased the nitrite levels also (Figure 4), generated by macrophages as compared to level generated by macrophages interacted with serovar Typhi cells, OMPs of serovar Typhi under normal conditions and with OMPs of serovar Typhi grown under acid stress (pH-4.5) conditions.

### Inflammatory potential of rFliC (*In-vivo*)

#### Mouse paw oedema test

**Figure 5:**
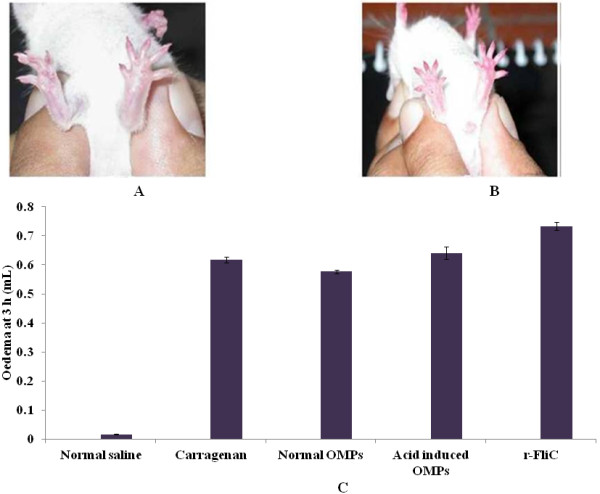
**Qualitative assessment of inflammation potential of rFliC. **Representative photographs of mice paw showing oedema (5A) Mouse injected with carrageenan (positive control) in the left foot paw showing oedema. (5B) Mouse showing marked inflammation in the left paw injected with rFliC. In each case, the right paw (injected with normal saline) serves as control. 5C shown, inflammatory potential of rFliC measured by plethysmometer scale . Inflammation caused by OMPs, carrageenan and normal saline were compared with rFliC.

The animals started developing oedema 20 min. after the injection and maximum oedema could be observed in all the groups at 3 h post injection. Inflammation was found to be the maximum in the mice injected with carrageenan (used as positive control) as compared to all the groups (Figure 5A &B). However, no inflammation was observed in the right paw of mice injected with normal saline which served as negative control. Amongst the remaining test groups, the magnitude of inflammation was observed to be the maximum with rFliC (Figure 5C) as compared to the normal and acid-induced OMPs.

### Hyperalgesic response

**Figure 6:**
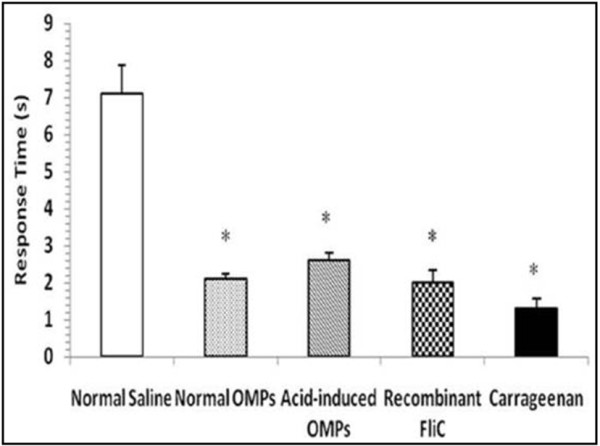
**Hyperalgesic response of the inflamed paw observed after 3 h in mice hind paw injected with different preparations. **Values are expressed as mean ±S.D. ^*^p<0.001 vs. control (normal saline) injected paw.

Thermal hyperalgesia depicts the response of mice with inflamed paws depending upon the time required for the withdrawal (flicking response) of the paw injected with different protein preparations (normal OMPs, Acid-induced OMPs and rFliC) (Figure 6). The flicking response observed was the fastest (i.e. time required for the paw withdrawal was the lowest) in case of the paw injected with rFliC indicating thermal hyperalgesic response of mouse to rFliC.

## Discussion

The gene encoding acid induced phenotype (AIP) in *Salmonella* was identified to be *fliC* gene in the previous study (Jindal et al. 2011). In the present report, serovar Typhi *fliC* gene was cloned, expressed and the recombinant protein was purified to assess the possible biological implications of this dynamic protein.

The *in-vivo* immunogenicity of rFliC was indicated by its ability to induce the production of antibodies and to react with them, which is in concordance with our previous study (Chander et al. 2004). Recognitions of FliC in the clinical isolates/samples using anti-FliC antibody also make the above mentioned observation more precise. Furthermore, the reactivity of FliC with the sera of typhoid patients (Widal positive) showed that anti-rFliC antibodies are produced in the serum during the natural course of infection. This observation is in agreement with the earlier study wherein acid-induced OMPs from serovar Typhi were found to be immunogenic *in-vivo* (Maripandi et al. 2010). Although the sera collected from patients suffering with pyrexia (but Widal test negative) did not react with this protein, sera from patients with non serovar Typhi bacteraemia along with a larger number of typhoid patient sera need to be screened to determine the specificity and the diagnostic utility of this protein.

Macrophages represent an important host defense mechanism. The ability of *Salmonella* to survive and replicate within these cells is thought to be one of the major determinants for *Salmonella* pathogenesis (Farr et al. 1986 and Schwan et al. 2000). There is considerable evidence that during *Salmonella*-induced infection, excessive generation of reactive oxygen species (ROS), like inflammatory molecules occurs which results into lipid peroxidation thereby causing damage to the host cell (Pacher et al. 2007). Many workers have used MDA level as an index of tissue damage since it is a stable product of oxidative attack of ROS on unsaturated fatty acids. From the present study also, it is interesting to observe that r-FliC alone could induce levels of MDA comparable to that with normal outer membrane proteins (OMPs) which in fact gives the combined effect of several outer membrane proteins, indicating thereby the higher inflammatory potential of FliC moiety.

Nitric oxide (NO), is another important signaling molecule that acts in many tissues to regulate a diverse range of physiological processes (Moncada et al. 1991). However, excessive amount of NO is potentially toxic and has been implicated in numerous pathological situations and chronic inflammation (Bogdan et al. 2001). Reactive nitrogen intermediates (RNI) such as nitrites are known to be the end products of labile nitric oxide and their quantification is regarded as an indicator of NO generation (Chanana et al. 2007b). Increased level of nitrite in presence of FliC observed in the present study appears to be attributed to TNF- α levels which is known for its potent immuno-stimulating activity of iNOS further increasing the NO level (Eaves-Pyles et al. 2001). This observation was also supported by earlier findings wherein flagellin has been reported to induce the expression of several inflammatory mediators including TNF-α and NO (Ciacci-Woolwine et al. 1998; Liaudet et al. 2003 and Zeng et al. 2003)]. Therefore, it is indicated that inflammatory potential of flagellin may be residing at least partly in the FliC subunit, a component of outer membrane of the bacteria. Earlier also, it has been reported that stress induced protein(s) do increase the levels of molecule such MDA and NO (Chander et al. 2006 and Chanana et al. 2007).

The ensuing inflammatory response of the intestinal mucosa has long been associated with *Salmonella* virulence (Valdez et al. 2009). It is known that OMPs have the potential to induce inflammatory reaction along with the release of cytokines (Chanana et al. 2005). In the present study also, the rFliC (an outer membrane phenotype) being the subunit of flagellar complex might have induced inflammation through the increased generation of pro-inflammatory mediators such as MDA and nitrite. Further, rFliC induced thermal hyperalgesia (flicking response) observed during inflammation may be due to the increased diacylglycerol levels. This in turn may activate protein kinase C (PKC) causing changes in pain perception (Pacher et al. 2007).

Therefore, the present study indicates the *in-vivo* immunogenic and inflammatory potential of flagellin (FliC) phenotype which is an integral part of flagellar motor complex, resides in the outer membrane of the serovar Typhi. Given the role of FliC in these biological implications, this phenotype may be another component of ATR, enhancing the virulence of the pathogen *in vivo*. Manipulating the expression of this protein may be helpful in formulating strategies for developing preventive intervention against *Salmonella* infection.

## Competing interests

The author(s) declare that they have no competing interests.

## Authors’ contributions

PR designed the experiments, analyzed the results and drafted the manuscript. RT provided help in developing the recombinant protein, AG designed the primer and helped in cloning experiment. GJ and SKP performed the experiments. All authors read and approved the final manuscript.
